# Evaluation of phenotypic and genotypic patterns of aminoglycoside resistance in the Gram-negative bacteria isolates collected from pediatric and general hospitals

**DOI:** 10.1186/s40348-022-00134-2

**Published:** 2022-02-04

**Authors:** Leila Azimi, Shahnaz Armin, Hossein Samadi Kafil, Nafiseh Abdollahi, Kiarash Ghazvini, Sepide Hasanzadeh, Shahram Shahraki Zahedani, Sedigheh Rafiei Tabatabaei, Fatemeh Fallah

**Affiliations:** 1 Pediatric Infections Research Center, Research Institute for Children’s Health, Shahid Beheshti University of Medical Sciences, P. Box, Tehran, 19857-17443 Iran; 2 Drug Applied Research Center, Faculty of Medicine, Tabriz University of Medical Sciences, Tabriz, Iran; 3 Department of Microbiology and Virology, Antimicrobial Resistance Research Center, Avicenna Research Institute, Faculty of Medicine, Mashhad University of Medical Sciences, Mashhad, Iran; 4 Department of Microbiology and Virology, Mashhad University of Medical Sciences, Mashhad, Iran; 5 Department of Medical Microbiology, School of Medicine, Zahedan University of Medical Sciences, Zahedan, Iran

**Keywords:** Aminoglycoside resistance, Gram-negative bacteria, Antibiotic resistance, Bacterial infection, Iran

## Abstract

The purpose of the current study was to evaluate the phenotypic and genotypic patterns of aminoglycoside resistance among the Gram-negative bacteria (GNB) isolates collected from pediatric and general hospitals in Iran. A total of 836 clinical isolates of GNB were collected from pediatric and general hospitals from January 2018 to the end of December 2019. The identification of bacterial isolates was performed by conventional biochemical tests. Susceptibility to aminoglycosides was evaluated by the disk diffusion method (DDM). The frequency of genes encoding aminoglycoside-modifying enzymes (AMEs) was screened by the PCR method via specific primers. Among all pediatric and general hospitals, the predominant GNB isolates were *Acinetobacter* spp. (*n* = 327) and *Escherichia coli* (*n* = 144). However, *E. coli* (*n* = 20/144; 13.9%) had the highest frequency in clinical samples collected from pediatrics. The DDM results showed that 64.3% of all GNB were resistant to all of the tested aminoglycoside agents. *Acinetobacter* spp. and *Klebsiella pneumoniae* with 93.6%, *Pseudomonas aeruginosa* with 93.4%, and *Enterobacter* spp. with 86.5% exhibited very high levels of resistance to gentamicin. Amikacin was the most effective antibiotic against *E. coli* isolates. In total, the results showed that the *aac (6')-Ib* gene with 59% had the highest frequency among genes encoding AMEs in GNB. The frequency of the surveyed aminoglycoside-modifying enzyme genes among all GNB was found as follows: *aph (3')-VIe* (48.7%), *aadA15* (38.6%), *aph (3')-Ia* (31.3%), *aph (3')-II* (14.4%), and *aph (6)* (2.6%). The obtained data demonstrated that the phenotypic and genotypic aminoglycoside resistance among GNB was quite high and it is possible that the resistance genes may frequently spread among clinical isolates of GNB.

**Supplementary Information:**

The online version contains supplementary material available at 10.1186/s40348-022-00134-2.

## Introduction 

Antimicrobial resistance is being increasingly recognized as a serious public health threat worldwide [1–4]. Antimicrobial resistance is highly noticeable among Gram-negative bacteria (GNB), and therefore, clinical isolates resistant to multiple or almost all available antibiotics have been consistently emerging [5, 6]. The three broad-spectrum classes of antimicrobial agents including β-lactams (especially β-lactam antibiotics and β-lactamase inhibitor combinations, cephalosporins, and carbapenems), aminoglycosides, and fluoroquinolones are the most common classes of antibiotics used to treat different infections caused by GNB [1, 7, 8]. Aminoglycosides as broad-spectrum antibiotics have an important role under clinical settings and are used for the treatment of severe and life-threatening hospital-acquired infections caused by GNB [9]. The aminoglycosides including tobramycin, gentamicin, and amikacin play a bactericidal role against a wide range of GNB such as *Acinetobacter baumannii* (*A. baumannii*), *Pseudomonas aeruginosa* (*P. aeruginosa*), *Escherichia coli* (*E. coli*), and *Klebsiella pneumoniae* (*K. pneumoniae*) [10, 11]. However, in recent years, resistance to aminoglycosides, especially its association with other antibiotic classes such as β-lactams and fluoroquinolones, has been increasingly reported. Resistance to aminoglycoside antibiotics is present virtually all over the world, particularly in Asia and Latin America [12]. The resistance mechanisms against aminoglycosides in GNB mainly result from the (1) production of aminoglycoside-modifying enzymes (AMEs), inactivating antibiotics classified in several families such as aminoglycoside nucleotidyltransferases (ANTs), aminoglycoside acetyltransferases (AACs), and aminoglycoside phosphoryltransferases (APHs); (2) methylation of 16S rRNA by a family of ribosomal methyltransferase enzymes; (3) mutation in the 30S ribosomal subunit; (4) active expulsion of antibiotics from the bacterial cells by efflux pumps; and (5) alteration of cell membrane permeability and reduction in intracellular concentration of aminoglycosides [13–15]. Among these factors, AMEs represent the most common mechanism by which clinical isolates of GNB and Gram-positive bacteria (GPB) can enzymatically modify the hydroxyl or amino groups of the drug, inhibiting their binding to ribosomes and hence allowing the bacteria to survive [16, 17]. According to the several condition such as partial immune system, neonates and children are a susceptible group to bacterial infections. Neonates can acquire bacteria from families or mothers within horizontally and vertically transmission ways, respectively. A*ntibiotic*-*resistant GNB can cause* severe infections in neonates and children and are considered the main concern for physicians [18].

In Iran, although some authors have reported a high prevalence rate of aminoglycoside resistance among the GNB isolated from clinical samples [19–21], the overall prevalence of aminoglycoside-resistant genes among clinical GNB isolates has not been determined widely. Therefore, the present study follows several objectives: (1) evaluation of the phenotypic resistance patterns of GNB; (2) determination of the frequency of common aminoglycoside-resistance genes including genes encoding AMEs; and (3) characterization of the correlation between aminoglycoside-resistance genes and the phenotypic resistance.

## Materials and methods

### Samples and bacterial isolation

The present study investigated the clinical isolates of GNB that were collected from pediatric and general hospitals throughout Iran during January 2018 to the end of December 2019 (Supplementary file).

Bacterial identification was carried out using conventional biochemical tests including growth on MacConkey (MAC) agar (Merck, Darmstadt, Germany), chocolate agar, and blood agar plates; evaluation of colony morphologies and Gram stain characteristics; oxidase test; catalase test; citrate utilization test; nitrate reduction test; indole production; motility; lactose fermentation; H_2_S production; urease activity; Methyl Red (MR) test; and Voges Proskauer (VP) test. After identification, all bacterial strains were preserved in Tryptic Soy Broth (Merck, Darmstadt, Germany) containing 20% glycerol at – 70 °C for further analysis.

### Antibiotic susceptibility testing

The susceptibility and resistance patterns of GNB to aminoglycoside agents were determined by the disk diffusion method (DDM) on Mueller Hinton agar medium (Merck, Darmstadt, Germany). The tested antimicrobial agents included amikacin (30 μg/disk), gentamicin (10 μg/disk), and tobramycin (30 μg/disk). The isolates resistant to at least one of these aminoglycoside agents were considered as aminoglycoside-resistant strain. The results of the DDM method were interpreted based on Clinical and Laboratory Standards Institute (CLSI) criteria. *E. coli* ATCC 25922 and *P. aeruginosa* ATCC 27853 were used as quality control strains in every test run.

### Molecular detection of aminoglycoside-resistant genes

Total genomic DNA was extracted from GNB isolates grown in Mueller-Hinton broth (Merck, Darmstadt, Germany) overnight at 37 °C. The bacterial cell pellets were resuspended in 250 μl of phosphate-buffered saline (PBS), and then, DNA extraction was performed using High Pure PCR Template Preparation Kit (Roche Diagnostics, Mannheim, Germany) in line with the manufacturer’s instructions. The purity and concentration of the extracted DNA were evaluated by Nanodrop (DeNovix Inc., USA). Extracted DNA was stored at – 80 °C for further analysis. The frequency of six main genes encoding AMEs including *aac (6')-Ib*, *aph (3')-VIe*, *aph (3')-II*, *aadA15*, *aph (3')-Ia*, and *aph (6)* was screened by polymerase chain reaction (PCR) method via specific primers. The sequence of the primers used for performing PCR is shown in Supplementary Table 1. The PCR reaction was performed on a thermal cycler (Eppendorf, Mastercycler Gradient; Eppendorf, Hamburg, Germany) under the following condition: 1 cycle at 95 °C for 5 min, followed by 30 cycles at 94 °C for 1 min, annealing at 56 °C, 57 °C, 60 °C, 63 °C, and 64 °C according to the specific temperature for each primer for 35–40 s, and then extension step at 72 °C for 1 min followed by a final extension step at 72 °C for 5 min. PCR products were analyzed on a 1.5% agarose gel, stained by DNA-safe stain (SinaClon, Tehran, Iran), and visualized under (UV) light (UVItec, Cambridge, UK).

### Statistical analysis

All data regarding the prevalence of isolated bacteria, resistance profile to aminoglycoside agents, and frequency of genes encoding AMEs were added to the statistical package SPSS v.23.0 (SPSS Inc., Chicago, IL, USA) and analyzed using descriptive statistical tests.

## Results

### Distribution of GNB in different clinical samples

From January 2018 to the end of December 2019, 836 GNB were collected from the pediatric and *general* hospitals in Iran. The hospital origin of all clinical samples is shown in the Supplementary file. In general, 836 different clinical samples were collected from Mofid children’s Hospital in Tehran, Iran. Mofid children’s Hospital is one of the most important educational and research centers in Tehran, the capital of Iran. The frequency of GNB among clinical samples collected from pediatrics was as follows: *E. coli* (*n* = 20/144; 13.9%), *Acinetobacter* spp. (*n* = 28/327;8.6%), *P. aeruginosa* (*n* = 8/136; 5.9%), *K. pneumoniae* (*n* = 20/140; 14.3%), and *Enterobacter* spp. (*n* = 25/89; 28%).

Among all pediatric and *general* hospitals, the isolated GNB included *Acinetobacter* spp. (*n* = 327; 39.1%), *E. coli* (*n* = 144; 17.2%), *K. pneumoniae* (*n* = 140; 16.5%), *P. aeruginosa* (*n* = 136; 16.3%), and *Enterobacter* spp. (89; 10.6%).

Among GNB, *Acinetobacter* spp. (*n* = 186; 56.9%) and *P. aeruginosa* (*n* = 55; 40.4%) were isolated frequently from tracheal samples. On the other hand, most isolates of *E. coli* (*n* = 119/144; 82.6%) and *K. pneumoniae* (*n* = 65/140; 46.4%) were isolated from urine samples. Moreover, *Enterobacter* spp. (*n* = 68/89; 76.4%) were isolated frequently from blood samples. The distribution of the isolated bacteria in clinical samples is shown in Supplementary Table 2.

### The frequency of GNB by age groups

The frequency of the GNB isolated from pediatric and general hospitals among different age groups is shown in Supplementary Table 3. In general, GNB had the highest and lowest frequency rates among patients in > 50-year and 5- to 14-year age groups, respectively. *P. aeruginosa* (59%), *Acinetobacter* spp. (53.7%), and *E. coli* (43.7%) showed the highest frequency in the age groups > 50 years. The results showed that *Acinetobacter* spp., *P. aeruginosa*, and *E. coli* isolates exhibited the lowest frequency in < 1-year, 1- to 4-year, and 5- to 14-year age groups. On the other hand, *K. pneumoniae* (27.1%) and *Enterobacter* spp. (25.8%) revealed the highest frequency in < 1-year age groups. Our analyses revealed that the frequency of *K. pneumoniae* (6.4%) among patients in the 5- to 14-year age group was low. Moreover, *Enterobacter* spp. had the lowest frequency in patients belonging to 15- to 29-year age groups.

### Antimicrobial susceptibility

#### Resistance rates of GNB to antimicrobials

The patterns of the resistance of the isolated GNB to aminoglycoside agents used are shown in Table 1. In total, the isolated GNB had the highest and lowest resistance rates to gentamicin (*n* = 758/836; 90.1%) and amikacin (*n* = 587/836; 70.2%), respectively. Among GNB, *Acinetobacter* spp. had the highest level of resistance to aminoglycoside agents and the resistance rates were found as follows: gentamicin (*n* = 306/327; 93.6%), tobramycin (*n* = 296/327; 90.5%), and amikacin (*n* = 295/327/90.2%). *E. coli* isolates represent the GNB that were significantly susceptible to the tested aminoglycosides with the following resistance rates: gentamicin (*n* = 117/144; 81.3%), tobramycin (*n* = 50/144; 34.7%), and amikacin (*n* = 39/144; 27%). Amikacin was the most effective antibiotic against *E. coli* isolates. Moreover, results showed that *K. pneumoniae* (*n* = 131/140; 93.6%), *P. aeruginosa* (*n* = 127/136; 93.4%), and *Enterobacter* spp. (*n* = 77/89; 86.5%) had the highest rates of resistance to gentamicin. Moreover, *P. aeruginosa* isolates had the same rate of resistance (*n* = 127/136; 93.4%) to tobramycin. In total, our results showed that 64.3% (*n* = 538/836) GNB including 103 *P. aeruginosa*, 296 *Acinetobacter* spp., 19 *E. coli*, 78 *K. pneumoniae*, and 42 *Enterobacter* spp. were resistant to all the three examined aminoglycoside antibiotics.

**Table 1:** Resistance patterns to aminoglycoside antibiotic agent among GNB in pediatric and general hospitals of Iran

Bacteria	Gentamicin			Amikacin			Tobramycin		
	S (%)	I (%)	R (%)	S (%)	I (%)	R (%)	S (%)	I (%)	R (%)
*Acinetobacter* spp. (*n* = 327)	14 (4.3)	7 (2.1)	306 (93.6)	28 (8.6)	4 (1.2)	295 (90.2)	27 (8.3)	4 (1.2)	296 (90.5)
*E. coli* (*n* = 144)	9 (6.2)	18 (12.5)	117 (81.3)	102 (70.8)	3 (2)	39 (27)	89 (61.8)	5 (3.5)	50 (34.7)
*K. pneumoniae* (*n* = 140)	7 (5)	2 (1.4)	131 (93.6)	54 (38.6)	2 (1.4)	84 (60)	19 (13.6)	0 (0)	121 (86.4)
*P. aeruginosa* (*n* = 136)	3 (2.2)	346 (4.4)	127 (93.4)	20 (14.2)	6 (4.4)	110 (81.4)	6 (4.5)	3 (2.2)	127 (93.3)
*Enterobacter* spp. (*n* = 89)	11 (12.4)	1 (1.1)	77 (86.5)	20 (22.5)	10 (11.2)	59 (66.3)	20 (22.3)	6 (6.4)	63 (71.3)
Total (836)	44 (5.3%)	34 (4%)	758 (90.1%)	224 (26.8%)	25 (3%)	587 (70.2%)	161 (19.3%)	18 (2.2%)	657 (78.6%)

*Abbreviations*: *S* susceptible, *I* intermediate, *R* resistance

#### Frequency of aminoglycoside-resistant genes among GNB

The current study evaluates the distribution of AME genes among phenotypically aminoglycoside-resistant GNB. The distribution of aminoglycoside-resistant genes among GNB is shown in Table 2. Results showed that *aac (6')-Ib* (*n* = 492/836; 59%) was the predominant aminoglycoside-modifying enzyme gene. The frequency of surveyed genes encoding AMEs among all GNB is given as follows: *aph (3')-VIe* (48.7%), *aadA15* (38.6%), *aph (3')-Ia* (31.3%), *aph (3')-II* (14.4%), and *aph (6)* (2.6%). The *aac (6')-Ib* (85.4%) and *aph (3')-Ia* (74.1%) genes had the highest frequency among the examined genes in *Enterobacter* spp. isolates. Moreover, the frequency of *aph (3')-IIb* (68%) and *aph (3')-VIe* (58%) genes was high in *P. aeruginosa* isolates. On the other hand, the *aadA15* gene (46.5%) was frequently detected among *K. pneumoniae* isolates. *aph (6)* gene was only detected in *E. coli* (11.8%) and *Acinetobacter* spp. (1.5%). The co-existence of aminoglycoside-resistant genes is shown in Table 3. Our analyses revealed that 8.2% (*n* = 27/327) of *Acinetobacter* isolates harbored simultaneously the *aac (6')-Ib, aph (3')-Ia*, *aadA15*, and *aph (3')-VIe* genes. Among *E. coli* isolates, 0.7% (*n* = 1/144) of the isolates harbored simultaneously *aac (6')-Ib*, *aph (6)*, *aadA15*, *aph (3')-II*, and *aph (3')-Ia* genes. The prevalence rates of the co-existence of five aminoglycoside resistance genes including *aac (6')-Ib*, *aph (3')-Ia*, *aadA15*, *aph (3')-II*, and *aph (3')-VIe* among *K. pneumoniae*, *P. aeruginosa*, and *Enterobacter* isolates were 0.7% (*n* = 1/140), 5.9% (*n* = 8/136), and 5.6% (*n* = 5/89), respectively.

**Table 2:** The distribution of aminoglycoside resistance genes among GNB in Iran

AME genes	*Acinetobacter* spp. (*n* = 327)	*E. coli* (*n* = 144)	*K. pneumoniae* (*n* = 140)	*P. aeruginosa* (*n* = 136)	*Enterobacter* spp. (*n* = 89)	Total
*aac (6')-Ib*	34.3% (112/327)	66% (95/144)	75.7% (106/140)	76.4% (104/136)	85.4% (76/89)	59% (493/836)
*aph (3')-VIe*	52.6% (172/327)	23.6% (34/144)	51.4% (72/140)	58% (79/136)	56.1% (50/89)	48.7% (407/836)
*aadA15*	37.9% (124/327)	43% (62/144)	46.5% (65/140)	36.8% (50/136)	24.7% (22/89)	38.6% (323/836)
*aph (3')-Ia*	35.5% (116/327)	18% (26/144)	20% (28/140)	19.1% (26/136)	74.1% (66/89)	31.3% (262/836)
*aph (3')-II*	4.6% (15/327)	1.3% (2/144)	2.1% (3/140)	68% (92/136)	9% (8/89)	14.4% (120/836)
*aph (6)*	1.5% (5/327)	11.8% (17/144)	0% (0/140)	0% (0/136)	0% (0/89)	2.6% (22/836)

**Table 3:** Aminoglycoside resistance gene profiles of the GNB in Iran

AME genes	*Acinetobacter* spp. (327) *N* (%)	*E. coli* (144) *N* (%)	*K. pneumoniae* (140) *N* (%)	*P. aeruginosa* (136) *N* (%)	*Enterobacter* spp. (89) *N* (%)
*aac (6')-Ib + aph (3')-Ia + aadA15 + aph (3')-II* + *aph (3')-VIe* (*n* = 14)	–	–	1/140 (0.7%)	8/136 (5.9%)	5/89 (5.6%)
*aac (6')-Ib + aph (6) + aadA15 + aph (3')-II* + *aph (3')-Ia* (*n* = 1)	–	1/144 (0.7%)	–	–	–
*aac (6')-Ib + aph (3')-Ia + aadA15 + aph (3')-VIe* (*n* = 52)	27/327 (8.2%)	5/144 (3.5%)	7/140 (5%)	3/136 (2.2%)	10/89 (11.2%)
*aac (6')-Ib + aph (3')-Ia + aph (3')-II* + *aph (3')-VIe* (*n* = 41)	9/327 (2.7%)	1/144 (0.7%)	7/140 (5%)	2/136 (1.5%)	22/89 (24.7%)
*aph (3')-Ia + aph (3')-II + aac (6')-Ib + aph (6)* (*n* = 29)	4/327 (1.2%)	1/144 (0.7%)	2/140 (1.4%)	–	22/89 (24.7%)
*aac (6')-Ib + aadA15 + aph (3')-Ia + aph (6)* (*n* = 6)	–	6/144 (4.2%)	–	–	–
*aac (6')-Ib + aph (6) + aadA15 + aph (3')-VIe* (*n* = 3)	–	3/144 (2%)	–	–	–
*aac (6')-Ib* + *aph (3')-VIe* (*n* = 57)	11/327 (3.4%)	10/144 (6.9%)	19/140 (13.6%)	11/136 (8%)	6/89 (6.7%)
*aadA15 + aac (6')-Ib* + *aph (3')-VIe* (*n* = 66)	24 (7.3%)	11/144 (7.6%)	25/140 (17.8%)	5/136 (3.7%)	1 (1.1%)
*aph (3')-Ia + aadA15 + aac (6')-Ib* (*n* = 25)	10/327 (3%)	7/144 (4.9%)	7/140 (5%)	–	1/89 (1.1%)
*aac (6')-Ib + aph (6) + aph (3')-VIe* (*n* = 12)	6/327 (1.8%)	–	–	5/136 (3.7%)	1/89 (1.1%)
*aac (6')-Ib + aph (6)* + *aph (3')-II* (*n* = 1)	1/327 (0.3%)	–	–	–	–
*aac (6')-Ib + aadA15* (*n* = 37)	4/327 (1.2%)	14/144 (9.7%)	15/140 (10.7%)	2/136 (1.5%)	2/89 (2.2)
*aph (3')-Ia + aadA15* (*n* = 15)	9/327 (2.7%)	4/144 (2.8%)	1/140 (0.7%)	–	1/89 (1.1%)
*aac (6')-Ib + aph (6)* (*n* = 3)	–	3/144 (2%)	–	–	–

The genotype profiles of bacterial isolates exhibiting resistance to the three detected antibiotics are shown in Table 4. Overall, 59.7% (*n* = 321/538) and 53.5% (*n* = 288/538) of all GNB that were resistant to all the three examined antibiotics harbored the *aph (3')-VIe* and *aac (6')-Ib* genes, respectively. Results showed that the frequency rates of *aac (6')-Ib* gene among *P. aeruginosa*, *Enterobacter* spp., *K. pneumoniae*, and *E. coli* were 95.1%, 71.4%, 69.2%, and 73.7%, respectively. Moreover, 60.8% of *Acinetobacter* spp. isolates were positive for the *aph (3')-VIe* gene.

**Table 4:** The genotype profiles for bacterial isolates showing resistance to three detected antibiotics

AME genes	*Acinetobacter* spp. (*n* = 296)	*E. coli* (*n* = 19)	*K. pneumoniae* (*n* = 78)	*P. aeruginosa* (*n* = 103)	*Enterobacter* spp. (*n* = 42)	Total
*aac (6')-Ib*	92/296 (31%)	14/19 (73.7%)	54/78 (69.2%)	98/103 (95.1%)	30/42 (71.4%)	288/538 (53.5%)
*aph (3')-VIe*	180/296 (60.8%)	8/19 (42.1%)	46/78 (59%)	65/103 (63.1%)	22/42 (52.4%)	321/538 (59.7%)
*aadA15*	120/296 (40.5%)	12/19 (63.1%)	42/78 (53.8%)	42/103 (40.8%)	17/42 (40.5%)	233/538 (43.3%)
*aph (3')-Ia*	109/296 (36.8%)	6/19 (31.6%)	19/78 (24.3%)	20/103 (19.4%)	29/42 (69%)	183/538 (34%)
*aph (3')-II*	10/296 (3.4%)	1/19 (5.3%)	2/78 (2.6%)	74/103 (71.8%)	5/42 (11.9%)	92/538 (17.1%)
*aph (6)*	2/296 (0.7%)	5/19 (26.3%)	0/78 (0%)	0/103 (0%)	0/42 (0%)	7/538 (1.3%)

## Discussion

In recent years, the incidence of both phenotypic and genotypic aminoglycoside resistance has been surging around the world [22, 23]. The corresponding high resistance rate can severely complicate combination therapy for aminoglycoside agents with broad-spectrum β-lactams including cephalosporins and carbapenems against severe Gram-negative infections, particularly in case of nosocomial infections [24, 25]. Since aminoglycoside agents are the first choice of clinicians for infection treatments [22] and given that aminoglycosides are the most commonly prescribed antimicrobial agents for treating serious GNB in Iran, an attempt is made here to characterize the aminoglycoside resistance by means of phenotypic and genotypic methods among five important GNB isolated from pediatric and *general* hospitals in Iran. The results of antimicrobial susceptibility screening revealed that about half of the GNB were fully resistant to at least one of the tested aminoglycosides, including gentamicin, tobramycin, and amikacin. Previously, resistance to gentamicin, amikacin, and tobramycin, which are considered as newer aminoglycosides with a broader spectrum of antibacterial activities, was generally found to be less common than resistance to older aminoglycosides such as streptomycin, kanamycin, and neomycin [26].

In the current study, approximately, more than 90% of *Acinetobacter* spp. exhibited resistance to all the tested aminoglycosides. In a previously published study by Aliakbarzade et al., 103 clinical *A. baumannii* strains were collected from Imam Reza Hospital of Tabriz University of Medical Sciences. They showed that *A. baumannii* strains were isolated from different clinical samples such as urine, sputum, tracheal secretion, bronchial lavage, wound, and blood. The findings of their study revealed that the rates of resistance to gentamicin, amikacin, and tobramycin were 86%, 81%, and 63%, respectively [27].

Compared to the above studies, we witnessed a significant increase in the number of aminoglycoside-resistant *Acinetobacter* isolates. In this study, the rate of resistance to gentamicin, amikacin, and tobramycin was 93.6%, 90.2%, and 90.5%, respectively. The high-level resistance to aminoglycoside is a serious issue for combination therapy of aminoglycoside with broad-spectrum β-lactams including carbapenems and cephalosporins against *Acinetobacter* infections [19]. According to our findings, in comparison to other tested aminoglycoside agents, amikacin caused less resistance in GNB, especially in *E. coli* isolates, and is still an extremely useful drug in treating severe *E. coli* infections. Several studies have pointed out that increased resistance against amikacin could be associated with the unrestricted use of this compound by some clinicians [28, 29].

In 2018, Nasiri et al. surveyed the molecular epidemiology of aminoglycoside resistance in clinical isolates of *K. pneumoniae*. They collected 177 *K. pneumoniae* strains from the patients admitted to intensive care units (ICUs) as well as infectious diseases, internal medicine, and surgery wards. *K. pneumoniae* strains were isolated from different clinical specimens such as urine, wound, sputum, trachea, and blood. The above authors reported that amikacin was a more active antimicrobial agent than other aminoglycosides toward clinical isolates of *K. pneumoniae* with a resistance rate of 61% [20]. Nevertheless, our findings show that the rate of resistance to amikacin in fact increased to 93.6% among *K. pneumoniae* isolates, compared to the mentioned studies. Overall, the high frequency of aminoglycoside-resistant GNB suggests that extensive use of these antimicrobial agents in clinical settings of Iran has led to the emergence of resistant isolates.

This study surveyed the prevalence of six main aminoglycoside-resistant genes in GNB. Results showed that a majority of aminoglycoside-resistant GNB (about three-quarters of isolates) harbored at least one AME gene. In total, the *aac (6')-Ib* (59%) and *aph (3')-VIe* (48.7%) genes were the most prevalent AME genes among all the aminoglycoside-resistant GNB. Related reports from different parts of the world have illustrated that *aac (6´)-II* and *ant (2´´)-I* genes are the most prevalent AME genes in Europe. Moreover, it has been revealed that *aph (3*′*)-VIe*, *ant (2´´)-I*, and *aac (6´)-I* genes have the highest frequency among AME genes in Korea [11, 30]. On the other hand, *aac (6´)-31/aadA15* and *aadA2* genes were also detected frequently in the GNB isolated from nosocomial infections in Mexico and Brazil [31, 32]. AMEs are the most important sources of aminoglycoside resistance among bacteria. The corresponding genes are highly mobile and can be transported by integrons, transposons, plasmids, and other transposable gene elements, often along with other resistant genes (such β-lactamases genes). As a matter of fact, the most threatening GNB acquire AME genes through horizontal gene transfer [33, 34].

In total, the *aac (6')* gene confers resistance to all of the aminoglycosides, except streptomycin. The *aph (3′)-VIe* was identified in *A. baumannii* and it conferred resistance to kanamycin, amikacin, neomycin, ribostamycin, paromomycin, butirosin, and isepamycin. The *aph (3′)-II* gene was described in the *P. aeruginosa* isolates. The *aph (3')-II* gene confers resistance to kanamycin, paromomycin, butirosin, neomycin, and ribostamycin. The *aadA1* gene remains resistant to streptomycin and spectinomycin. Moreover, the *aph (3')-Ia* and *aph (6)* genes correspond to the resistance to kanamycin and tobramycin, respectively [17, 35, 36].

In Iran, a high prevalence rate of AMEs was previously reported in GNBs such as *P. aeruginosa*, *A. baumannii*, and *K. pneumoniae* [20, 37, 38]. The *aph (3')-VIe* was the most common AME in *Acinetobacter* isolates (52.6%), followed by *aadA15* (37.9%), *aph (3')-Ia* (35.5%), and *aac (6')-Ib* (34.3%). In a study conducted by Aghazadeh et al*.* in Iran, *aph(3')-VIe* and *aph(3')-II* were the most prevalent AME genes in *A. baumannii* with prevalence rates of 90.6% and 61.8%, respectively [39]. In another study in Iran, Asadollahi et al. reported that AME genes including *aadA12*, *aacC1*, and *aadB* were the most prevalent ones among *A. baumannii* [40]. Altogether, these data indicate that *aph (3')-VIe* and *aadA15* genes contribute to aminoglycoside resistance among clinical isolates of *A. baumannii* in different geographic locations of Iran. Our results were similar to those found by Nasiri et al. in Iran who reported *aac(6′)* as the most dominant AME among clinical isolates of *K. pneumoniae* [20]. However, Ghotaslou et al. previously reported that *ant(3″)-Ia* and *aph(3″)-Ib* were the most prevalent AME genes in *Enterobacteriaceae* isolates in the northwest of Iran with frequency rates of 35.9% and 30.5%, respectively [14]. In another research by Soleimani et al., *aac (3)-IIa* and *ant(2″)-Ia* genes were identified as the most common AMEs in uropathogenic *E. coli* isolated from an Iranian hospital [41]. These data suggested that various reasons such as diversity of specimen type, geographic regions, sample size, bacterial sources, usage of antibiotics, and applied detecting methods would affect the distribution patterns of AME. Liang et al. previously reported that *aac (3)-II*, *aac (6′)-Ib*, and *ant (3″)-I* genes were the most common AME genes in *K. pneumoniae* isolates in China [42]. In Norway, Lindemann et al. indicated that the majority of *E. coli* and *K. pneumoniae* isolates in their study harbored *aac(3)-IIa* and *aac(6′)-Ib* genes [42]. The significant variation of the results may be attributed to geographical factors. Regarding *P. aeruginosa* isolates, we found *aac (6')-Ib* and *aph (3')-II* as the most prevalent AME genes. These findings are consistent with those reported by Aghazadeh et al. in Tabriz, Iran [39]. In general, results demonstrated that more than 90% of GNB were resistant to one of the antibiotics. However, the results of the molecular method revealed that 59% of GNB harbored the *aac (6')-Ib* gene. Our analyses revealed that 78% of GNB were positive for at least one of the examined genes.

The limitation of this study is that we just sequenced one positive sample of each gene. For sequencing the *aac (6')-Ib*, *aph (3')-II*, *aph (6)*, *aadA15*, and *aph (3')-Ia* genes, we used a positive sample in *E. coli* isolates. Moreover, one positive sample among *A. baumannii* isolates was used for *aph (3')-VIe* gene sequencing. This limitation was due to the budget limitation. In the analyzed sequences, all taxon affiliation was performed automatically by GenBank in the sequence submission process.

## Conclusion

The data obtained in this study indicated that resistance to aminoglycoside in Iran was high and AME genes frequently spread among clinical GNB isolates. Therefore, there are enough reasons to assume that the increasing complexity of aminoglycoside resistance mechanisms is associated with the high complexity of aminoglycoside usage in Iran. However, constant monitoring and surveillance of aminoglycoside resistance, antimicrobials, and consumption can improve the antibiotics prescription regimen and prevent the spread of these resistant bacteria in communities and hospital settings.

## Supplementary Information

**Additional file 1: Supplementary information.** The hospital origin of all clinical samples.

**Additional file 2: Supplementary Table 1*.*** Primers used for the detection of genes encoding AMEs.

**Additional file 3: Supplementary Table 2.** Frequency of GNB among different clinical samples in pediatric and general hospitals of Iran.

**Additional file 4: Supplementary Table 3*.*** The frequency of GNB isolated from clinical samples by different age groups.

## Data Availability

All data generated or analyzed during this study are included in this published article.

## Abbreviations

GNB: Gram-negative bacteria
GPB: Gram-positive bacteria
AMEs: Aminoglycoside-modifying enzymes
DDM: Disk diffusion method
ANTs: Aminoglycoside nucleotidyltransferases
AACs: Aminoglycoside acetyltransferases
APHs: Aminoglycoside phosphoryltransferases
MAC: MacConkey
MR: Methyl Red
VP: Voges Proskauer
CLSI: Clinical and Laboratory Standards Institute
