# Supplementary material for: Evaluation of phenotypic and genotypic patterns of aminoglycoside resistance in the Gram-negative bacteria isolates collected from pediatric and general hospitals
Source: Mol Cell Pediatr. 2022 Feb 4;9:2. doi: 10.1186/s40348-022-00134-2 (PMC8816979; doi:10.1186/s40348-022-00134-2)
Supplement: Supplementary file 1 — Additional file 1: Supplementary information. The hospital origin of all clinical samples. [file 40348_2022_134_MOESM1_ESM.docx]

**Supplementary information:**

**The hospital origin of all clinical samples.**

In general, the frequency of isolated GNB among different cities of Iran was as follows:

**Tehran province**:

**Mofid hospital**: *E. coli* (n = 20/144;13.9%), *Acinetobacter* spp. (n = 28/327;8.6%), *P. aeruginosa* (n = 8/136; 5.9%), *K. pneumoniae* (n = 20/140; 14.3%), and *Enterobacter* spp. (n = 25/89;28%).

**Milad hospital**: *E. coli* (n = 14/144;9.7%), *Acinetobacter* spp. (n = 30/327;9.2%), *P. aeruginosa* (n = 17/136; 12.5%), *K. pneumoniae* (n = 22/140; 15.7%), and *Enterobacter* spp. (n = 15/89;16.8%).

**Ahvaz province**: *E. coli* (n = 10/144;7%), *Acinetobacter* spp. (n = 2/327;0.6%), *P. aeruginosa* (n = 0/136; 0%), *K. pneumoniae* (n = 8/140; 5.7%), and *Enterobacter* spp. (n = 4/89;4.5%).

**Hamadan province***: E. coli* (n = 9/144;6.2%), *Acinetobacter* spp. (n = 60/327;18.3%), *P. aeruginosa* (n = 26/136; 19.1%), *K. pneumoniae* (n = 18/140; 12.9%), and *Enterobacter* spp. (n = 1/89;1.1%).

**Isfahan province**: *E. coli* (n = 13/144;9%), *Acinetobacter* spp. (n = 54/327;16.5%), *P. aeruginosa* (n = 26/136; 19.1%), *K. pneumoniae* (n = 25/140; 17.9%), and *Enterobacter* spp. (n = 10/89;11.2%).

**Tabriz province**: *E. coli* (n = 10/144;7%), *Acinetobacter* spp. (n = 63/327;19.3%), *P. aeruginosa* (n = 25/136; 18.4%), *K. pneumoniae* (n = 8/140; 5.7%), and *Enterobacter* spp. (n = 11/89;12.4%).

**Zahedan province**: *E. coli* (n = 25/144;17.4%), *Acinetobacter* spp. (n = 34/327;10.4%), *P. aeruginosa* (n = 5/136; 3.7%), *K. pneumoniae* (n = 11/140; 7.8%), and *Enterobacter* spp. (n = 7/89;7.9%).

**Gorgan province**: *E. coli* (n = 8/144;5.5%), *Acinetobacter* spp. (n = 0/327;0%), *P. aeruginosa* (n = 0/136; 0%), *K. pneumoniae* (n = 0/140;0%), and *Enterobacter* spp. (n = 2/89;2.2%).

**Mashhad province**: *E. coli* (n = 16/144;11.1%), *Acinetobacter* spp. (n = 23/327;7%), *P. aeruginosa* (n = 17/136; 12.5%), *K. pneumoniae* (n = 9/140; 6.4%), and *Enterobacter* spp. (n = 8/89;9%).

**Sanandaj province:** *E. coli* (n = 19/144;13.2%), *Acinetobacter* spp. (n = 33/327;10%), *P. aeruginosa* (n = 12/136; 8.8%), *K. pneumoniae* (n = 19/140; 13.6%), and *Enterobacter* spp. (n = 6/89;6.7%).

**The frequency of GNB in different wards of hospitals was as follows:**

***E. coli* (n = 144)**

Respiratory (2), Emergency (20), ICU (16), Transplant (6), Neurology (3), Renal unit (14), Gastroenterology (5), Surgery (10), Orthopedics (5), Infectious (1), Urology (11), Internal (6), Thorax (2), General (21), Hematology (2), Dialysis (3), NICU (4), PICU (13).

***Acinetobacter* spp. (n = 327)**

General (7), Neurology (13), Respiratory (19), Emergency (17), ICU (150), Transplant (6), Renal unit (5), Gastroenterology (3), Surgery (15), Orthopedics (4), Infectious (18), Urology (4), Internal (10), Trauma (12), Thorax (2), Burn (6), Dialysis (1), NICU (6), PICU (29).

***P. aeruginosa* (n = 136)**

Gastroenterology (5), Urology (1), Neurology (4), Internal (14), Respiratory (2), Emergency (8), ICU (63), Renal unit (1), Infectious (2), Surgery (11), Orthopedics (2), General (3), Trauma (3), Burn (13), PICU (4).

***K. pneumoniae* (n = 140)**

General (12), Surgery (16), Emergency (10), ICU (20), Dialysis (1), Transplant (8), PICU (22), Neurology (2), Renal unit (12), Infectious (11), Urology (5), Internal (6), Trauma (5), NICU (9), Rheumatology (1).

***Enterobacter* spp. (n = 89)**

Respiratory (4), Emergency (16), ICU (11), Transplant (3), Neurology (4), Renal unit (4), Gastroenterology (5), Surgery (7), Orthopedics (2), Infectious (7), Urology (3), Internal (5), General (9), Hematology (2), Dialysis (2), PICU (5).
