# Supplementary material for: Evaluation of phenotypic and genotypic patterns of aminoglycoside resistance in the Gram-negative bacteria isolates collected from pediatric and general hospitals
Source: Mol Cell Pediatr. 2022 Feb 4;9:2. doi: 10.1186/s40348-022-00134-2 (PMC8816979; doi:10.1186/s40348-022-00134-2)
Supplement: Supplementary file 2 — Additional file 2: Supplementary Table 1. Primers used for the detection of genes encoding AMEs. [file 40348_2022_134_MOESM2_ESM.docx]

Supplementary Table 1. Primers used for the detection of genes encoding AMEs.

| Target | Sequence (5′→3′) | Annealing temperature (C°) | Product size  (bp) | Reference |
| --- | --- | --- | --- | --- |
| *aac (6')-Ib* | F: TTGCGATGCTCTATGAGTGGCTA | 63 | 482 | [1] |
|  | R: CTCGAATGCCTGGCGTGTTT |  |  |  |
| *aph (3')-II* | F: ATGCATGATGCAGCCACCTCC | 64 | 807 | [2] |
|  | R: CTAGAAGAACTCGTCCAATAGCCT |  |  |  |
| *aph* (3')-*VIe* | F: AGCGAAAATGTTGAGTTGGCT | 57 | 339 | [3] |
|  | R: TCCGTGATATCGCCATGAGA |  |  |  |
| *aadA15* | F: GTGGATGGCGGCCTGAAGCC | 63 | 527 | [4] |
|  | R: AATGCCCAGTCGGCAGCG |  |  |  |
| *aph (3')-Ia* | F: AAACGTCTTGCTCGAGGC | 56 | 461 | [5] |
|  | R: CAAACCGTTATTCATTCGTGA |  |  |  |
| *aph (6)* | F: GAGCGCACCTTCGACTATGC | 63 | 248 | [2] |
|  | R: GCCATGGCGTTTACGGCCAG |  |  |  |

1. Park CH, Robicsek A, Jacoby GA, Sahm D, Hooper DC (2006) Prevalence in the United States of aac(6')-Ib-cr encoding a ciprofloxacin-modifying enzyme. Antimicrobial agents and chemotherapy 50 (11):3953-3955. doi:10.1128/AAC.00915-06

2. Nie L, Lv Y, Yuan M, Hu X, Nie T, Yang X, Li G, Pang J, Zhang J, Li C, Wang X, You X (2014) Genetic basis of high level aminoglycoside resistance in Acinetobacter baumannii from Beijing, China. Acta pharmaceutica Sinica B 4 (4):295-300. doi:10.1016/j.apsb.2014.06.004

3. Khoshnood S, Eslami G, Hashemi A, Bahramian A, Heidary M, Yousefi N, Mohammdi F, Gholami M (2017) Distribution of Aminoglycoside Resistance Genes Among Acinetobacter baumannii Strains Isolated From Burn Patients in Tehran, Iran. 5 (3):e57263. doi:10.5812/pedinfect.57263

4. Zishiri OT, Mkhize N, Mukaratirwa S (2016) Prevalence of virulence and antimicrobial resistance genes in Salmonella spp. isolated from commercial chickens and human clinical isolates from South Africa and Brazil. The Onderstepoort journal of veterinary research 83 (1):a1067-a1067. doi:10.4102/ojvr.v83i1.1067

5. Gebreyes WA, Thakur S (2005) Multidrug-resistant Salmonella enterica serovar Muenchen from pigs and humans and potential interserovar transfer of antimicrobial resistance. Antimicrobial agents and chemotherapy 49 (2):503-511. doi:10.1128/AAC.49.2.503-511.2005
