# Supplementary material for: Evaluation of phenotypic and genotypic patterns of aminoglycoside resistance in the Gram-negative bacteria isolates collected from pediatric and general hospitals
Source: Mol Cell Pediatr. 2022 Feb 4;9:2. doi: 10.1186/s40348-022-00134-2 (PMC8816979; doi:10.1186/s40348-022-00134-2)
Supplement: Supplementary file 3 — Additional file 3: Supplementary Table 2. Frequency of GNB among different clinical samples in pediatric and general hospitals of Iran. [file 40348_2022_134_MOESM3_ESM.docx]

Supplementary Table 2. Frequency of GNB among different clinical samples in pediatric and general hospitals of Iran.

| Source |  | Bacteria (n) | | | |
| --- | --- | --- | --- | --- | --- |
|  | *Acinetobacter* spp.  N (%) | *P. aeruginosa*  N (%) | *E. coli*  N (%) | *K. pneumoniae*  N (%) | *Enterobacter* spp.  N (%) |
| Blood  Wound  Secretion  Urine  CSF  Bronchial  Catheter  Tracheal  Eye  Septum  Abscess  Peritoneal  Bone  Tissue  Total | 34 (10.4) | 12 (8.8) | 2 (1.4) | 17 (12.1) | 68 (76.4) |
|  | 28 (8.6) | 21 (15.4) | 11 (7.6) | 4 (2.8%) | 3 (3.4) |
|  | 13 (3.4) | 3 (2.2) | 3 (2) | 2 (1.4%) | 1 (1.1) |
|  | 13 (3.4) | 27 (19.8) | 119 (82.6) | 65 (46.4%) | 10 (11.2) |
|  | 8 (2.4) | 4 (2.9) | 2 (1.4) | 4 (2.8%) | 0 (0) |
|  | 24 (7.3) | 4 (2.9) | 1 (0.7) | 5 (3.6%) | 3 (3.4) |
|  | 4 (1.2) | 5 (3.7) | 0 (0) | 7 (5%) | 0 (0) |
|  | 186 (56.9) | 55 (40.4) | 1 (0.7) | 24 (17.1%) | 2 (2.2) |
|  | 0 (0) | 0 (0) | 1 (0.7) | 3 (2.1%) | 1 (1.1) |
|  | 16 (4.8) | 4 (2.9) | 0 (0) | 4 (2.8%) | 0 (0) |
|  | 0 (0) | 1 (0.7) | 2 (1.4) | 4 (2.8%) | 1 (1.1) |
|  | 1(0.3) | 0 (0) | 1 (0.7) | 0 (0%) | 0 (0) |
|  | 0 (0) | 0 (0) | 1 (0.7) | 0 (0%) | 0 (0) |
|  | 0 (0) | 0 (0) | 0 (0) | 1 (7.1%) | 0 (0) |
|  | 327 (100) | 136 (100) | 144 (100) | 140 (100) | 89 (100) |
