# Supplementary material for: Evaluation of phenotypic and genotypic patterns of aminoglycoside resistance in the Gram-negative bacteria isolates collected from pediatric and general hospitals
Source: Mol Cell Pediatr. 2022 Feb 4;9:2. doi: 10.1186/s40348-022-00134-2 (PMC8816979; doi:10.1186/s40348-022-00134-2)
Supplement: Supplementary file 4 — Additional file 4: Supplementary Table 3. The frequency of GNB isolated from clinical samples by different age groups. [file 40348_2022_134_MOESM4_ESM.docx]

| Bacteria | Age groups | | | | | | Total |
| --- | --- | --- | --- | --- | --- | --- | --- |
|  | <1YR | 1-4YR | 5-14YR | 15-29YR | 30-50YR | >50YR |  |
| *Acinetobacter* spp. | 5 (3.7%) | 6 (4.4%) | 7 (5.1%) | 17 (12.5%) | 28 (20.6%) | 73 (53.7%) | 136 |
| *P. aeruginosa* | 13 (3.4%) | 10 (3%) | 15 (4.6%) | 41 (12.5%) | 55 (16.8%) | 193 (59%) | 327 |
| *E. coli* | 12 (8.3%) | 19 (13.2%) | 7 (4.9%) | 10 (7%) | 33 (23%) | 63 (43.7%) | 144 |
| *K. pneumoniae* | 38 (27.1%) | 11 (7.9%) | 9 (6.4%) | 17 (12.1%) | 27 (19.3%) | 38 (27.1%) | 140 |
| *Enterobacter* spp. | 23 (25.8%) | 18 (20.2%) | 13 (14.6%) | 5 (5.6%) | 10 (11.2%) | 20 (22.5%) | 89 |
| Total | 91 (10.9%) | 64 (7.6%) | 51 (6.1%) | 90 (10.8%) | 153 (18.3%) | 387 (46.3%) | 836 |

Supplementary Table 3. The frequency of GNB isolated from clinical samples by different age groups.
